# Supplementary material for: Patient-specific cancer genes contribute to recurrently perturbed pathways and establish therapeutic vulnerabilities in esophageal adenocarcinoma
Source: Nat Commun. 2019 Jul 15;10:3101. doi: 10.1038/s41467-019-10898-3 (PMC6629660; doi:10.1038/s41467-019-10898-3)
Supplement: Supplementary file 1 — Supplementary Information [file 41467_2019_10898_MOESM1_ESM.pdf]

**Supplementary Information**

# Patient-specific cancer genes contribute to recurrently perturbed pathways and establish therapeutic vulnerabilities in esophageal adenocarcinoma.

Mourikis, Benedetti, Foxall et al.

**Supplementary Note 1.** Description of the sysSVM algorithm

## Motivation and rationale

sysSVM (systems-level Support Vector Machine) is an algorithm to predict cancer genes in individual patients based on features derived from their molecular and systems-level properties. sysSVM builds on our previous efforts to identify novel cancer genes in each sample independently as opposed to focussing on recurrently altered genes across sample cohorts[1]. An approach based on sample-specific analysis is advantageous in the presence of highly heterogeneous cancers (such as esophageal adenocarcinoma) where the mutational landscape is highly variable across samples and recurrently altered genes are rare. sysSVM replaces the comparison of frequency of gene alterations across samples with the analysis of gene properties in individual samples, following the principle that genes contributing to cancer share similar properties.

## sysSVM implementation

While several sources of true positive observations (*i.e.* known cancer genes) are available, a set of true negative observations (*i.e.* non-cancer genes) is difficult to assemble. One possibility would be to rely on known false positives of driver prediction approaches[2]. However, these genes often have distinct properties (i.e. they are long genes with a biased sequence composition) and this would bias the predictor as they are not representatives of all non-cancer genes. To overcome this problem, sysSVM implements a one-class support vector machine for novelty detection that models the density of the data in the input feature space[3]. The strategy of a one-class SVM is to map the data to the feature space corresponding to the chosen kernel and separate them from the origin with the maximum margin but without using a negative set[3]. As a positive set, sysSVM uses all known cancer genes with damaging alterations in the sample cohort under study and it is based on several steps as detailed below and in Figure 1A. Training and prediction are done using linear, radial, sigmoid and polynomial kernels and, after identifying the best model in each kernel, genes are ranked in each sample individually using a combined score. sysSVM is implemented in R using the e1071 package[4].

**Step 1: Feature mapping**

Step 1 of sysSVM consists of mapping 34 features to all genes altered in the sample cohort under study. Ten of the 34 features derive from molecular properties and 24 features derive from systems-level properties of known cancer genes (Supplementary Table 1). These 34 features are used to define the regions of the feature space where the known cancer genes reside. Twenty-two of them are categorical and 12 are continuous variables (Supplementary Table 1).

**Step 2: Model selection**

In step 2 of sysSVM, a grid search is performed to optimise the parameters used in each kernel:

1) *nu* (all kernels), representing the upper bound on the fraction of outliers (*i.e.* training genes left outside the estimated region) and the lower bound on the fraction of support vectors. Values for *nu* range from 0.05 to 0.9 with a step of 0.05 for a total of 18 values;

2) *gamma* (radial, sigmoid, and polynomial kernels), accounting for the influence of individual training points in the final model and defined as:

$$\gamma = 2^x, \text{ where } x \in \{-7, -6, \dots, 4\} \quad (1)$$

for a total of 12 values;

3) *degree* (polynomial kernel), representing the degree of the polynomial kernel function with three possible values (3, 4, 9);

The grid search results in 18 combinations of parameters for the linear kernel, 216 combinations for the radial and sigmoid kernels and 648 combinations for the polynomial kernel, for a total of 1098 combinations.

To identify the best combination of parameters for each kernel, a user-defined number of iterations of three-fold cross validations is performed (default = 10,000). At each iteration, the genes of the training set are randomly split into two subsets, one used for training (2/3 of the genes) and one as a test set (1/3 of the genes). Predictions are performed on the test set and the sensitivity of each set of parameters is computed. The distribution of sensitivity every n iterations (default = 100) is derived. The least variant model among the top five most sensitive models in each kernel (considering the mean sensitivity) is chosen as the best model for that kernel. To account for the effect of increasing number of cross validation iterations, at each increment of n cross

validations (default = 100), the selection of best models takes into account all previous cross-validation iterations.

To account for the effect of the order of iterations, this cumulative assessment is repeated a number of times (default = 5) where the iterations of cross validation are randomly reordered. This produces m sets of best models (from a default of 5 re-orderings of 100 increments, m = 500).

**Step 3: Training and prediction**

In step 3, all m sets of best models identified in step 2 are used for training using the whole training set. Cancer genes are then predicted in individual samples from all genes with damaging alterations (excluding the known cancer genes used for training) using each best model. To combine the resulting four sets of predictions (one from each kernel), a combined score ($S_{gs}$) for each altered gene *(g)* in each sample *(s)* is derived. $S_{gs}$ takes into account the similarity of the features of gene *(g)* to those of the training set by summing up its rank in sample *(s)* in each kernel (*i*), ($R_{igs}$). $R_{igs}$ is derived by sorting the decision values (indicative of the distance of the gene from the decision boundary that separates positive from negative sets) of kernel (*i*) within sample *(s)* so that high decision values correspond to top scoring genes. $S_{gs}$ then corrects for the total number of altered genes in that sample and for the sensitivity of each kernel and applies a normalisation factor to scale the resulting value between 0 and 1:

$$S_{gs} = \frac{\sum_{i=1}^{4} \left(-log_{10}\left(\frac{R_{igs}}{N_s}\right) \times BMS_i\right)}{4 \times log_{10}(N_s)} \qquad (2)$$

where $N_s$ is the number of altered genes in sample *(s)*; $R_{igs}$ is the rank of gene (*g*) in sample *(s)* and kernel (*i*); and $BMS_i$ is the sensitivity of the best model in kernel (*i*).

Genes are ranked using $S_{gs}$ and the top k genes in each patient (default top 10) are retained for further comparison. The m sets of best models produce m lists of top k genes (default = 500). The most frequent list of top k genes overall is selected as the final list of predicted cancer genes.

**Supplementary Figure 1.** Pairwise correlation between 34 features for classification

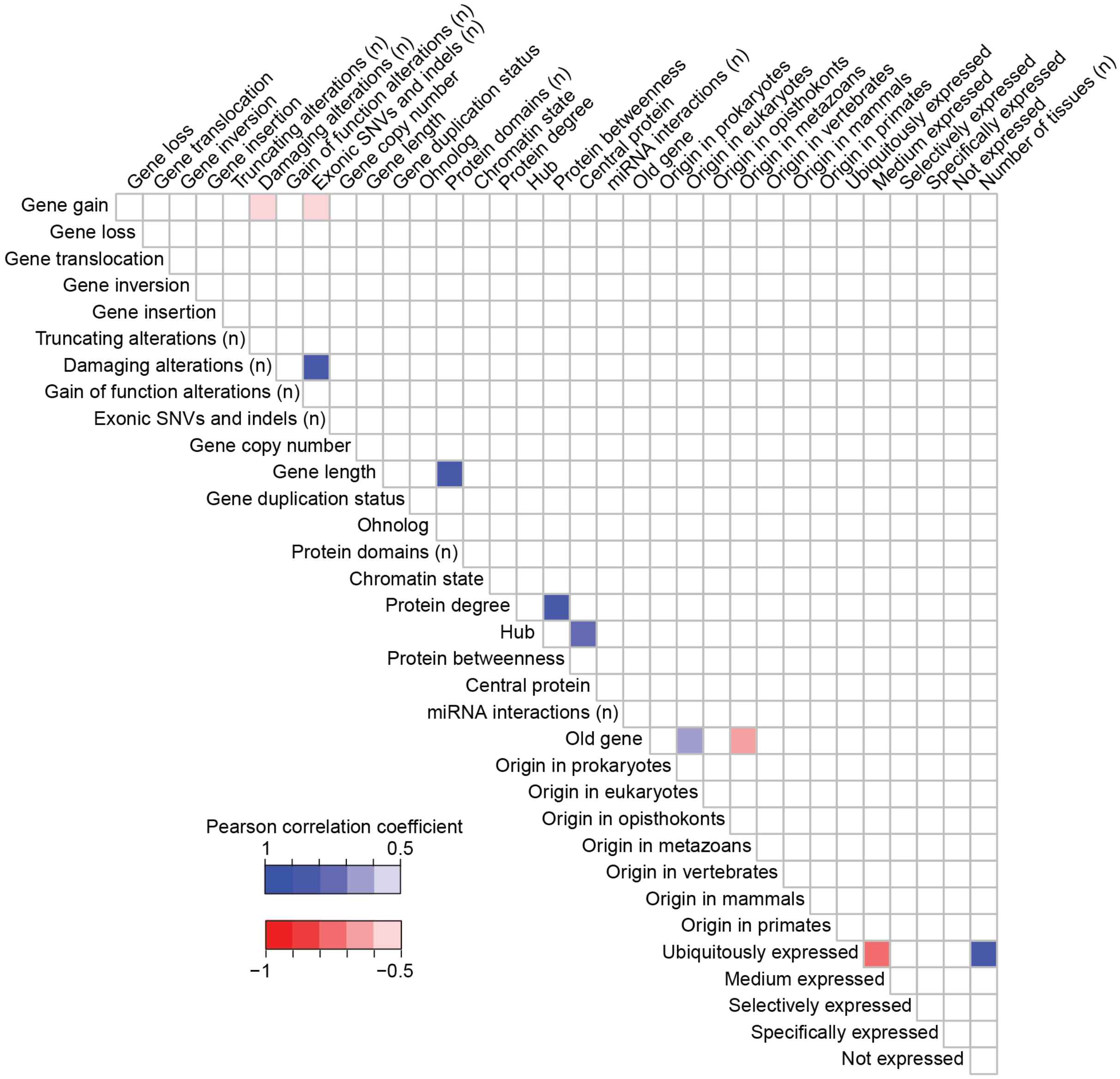


**Legend:** For each pair of features, a Pearson correlation coefficient was measured considering all values of 17,078 genes. Only coefficient values between [-0.5, 0.5] and with FDR≤0.05 are shown. Positive correlations were observed between protein degree and betweenness as well as between highly connected (hub) and central proteins. These are known relationships between properties of nodes in a network. Other positive correlations were observed between the number of damaging alterations and the total number of exonic SNVs and indels; between gene length and protein domains; and between genes ubiquitously expressed and the number of tissues where they are expressed. We decided to keep all these features because they are complementary in their description of gene properties.

**Supplementary Figure 2.** tSNE plots of 34 properties of known cancer genes

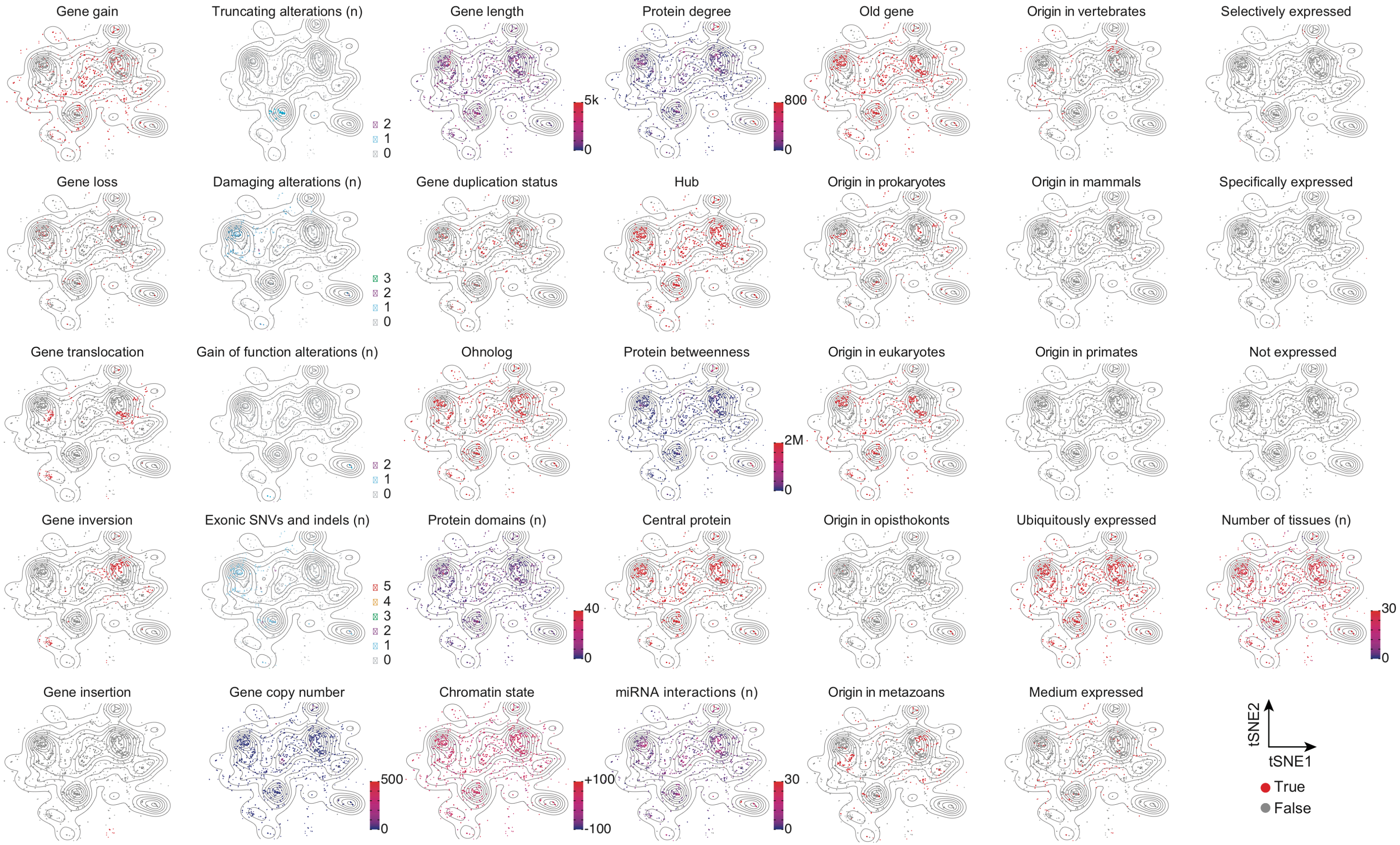

**Legend:** For each property, a 2-D map of the high-dimensional data was rebuilt for the 476 known cancer genes altered 4,091 times in the cohort of 261 EACs. Black curves represent the density of known cancer genes. For continuous or multi-value variables (truncating alterations, non-truncating damaging alterations, gain of function alterations, exonic SNVs and indels, gene copy number, gene length, protein domains, chromatin state, protein degree, protein betweenness, miRNA interactions, tissues where the gene is expressed) a colour code is reported. For categorical variables (gene gain, gene loss, gene translocation, gene inversion, gene insertion, gene duplication status, ohnolog, hub, central protein, old gene, origin in prokaryotes, origin in single cell eukaryotes, origin in opisthokonts, origin in metazoans, origin in vertebrates, origin in mammals, origin in primates, ubiquitously expressed, medium expressed, selectively expressed, specifically expressed, not expressed) genes are labelled according to whether they have (red) or not (grey) that property.

## **Supplementary Figure 3.** sysSVM validation

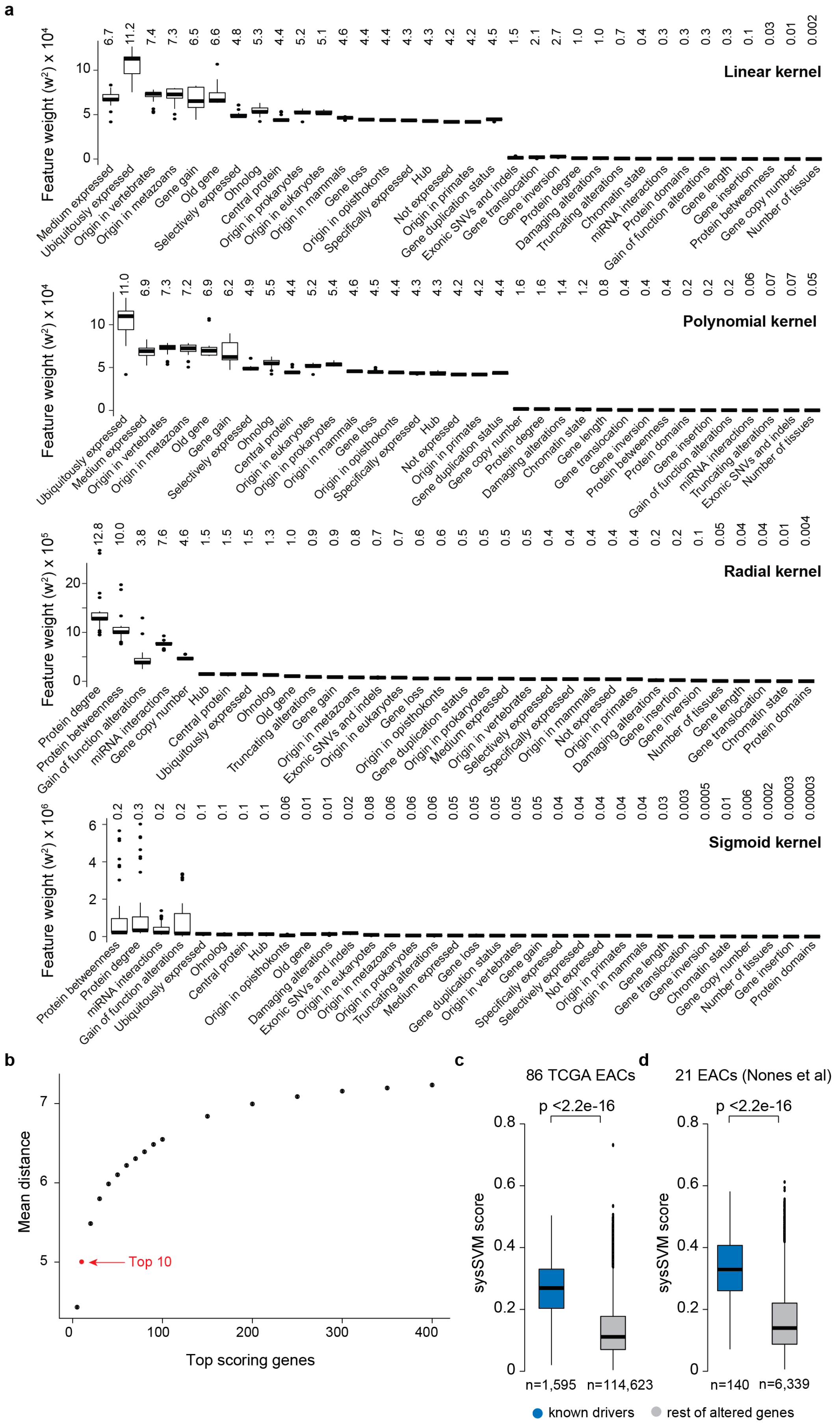

**Legend: a.** Feature ranking using Recursive Feature Elimination (RFE)[5]. For each kernel, the distribution of the squared weight of each feature across 34 iterations is shown. The median value of the distribution is shown above each box.

Features are sorted according to their rank, measured in terms of the iteration where each feature was eliminated (*i.e.* the leftmost feature in each plot was the last to be eliminated in the corresponding kernel). This is the reason why the top-ranking features do not necessarily have the highest median squared weight. **b.** Average distance from the center of the highest-density regions of known cancer genes for different score thresholds to define helper genes. For each gene set, the distance of each helper gene from the center was calculated and the mean of the distribution across the set was derived. Comparison of sysSVM scores between known drivers and the rest of altered genes for 86 EACs from TCGA (**c**) and 21 EACs from Nones et al., 2014[6]. (**d**). Starting from all altered genes, known drivers were identified as described in the Methods. All genes that are not expressed in healthy esophagus were removed from both gene sets. Distributions were compared using two tailed Wilcoxon rank-sum test. Lower and upper hinges and middle line of boxplots correspond to 25$^{th}$, 75th and 50$^{th}$ percentiles. Upper and lower whiskers extend less than 1.5 times the interquartile range.

**Supplementary Figure 4.** Co-amplifications and features of known cancer drivers

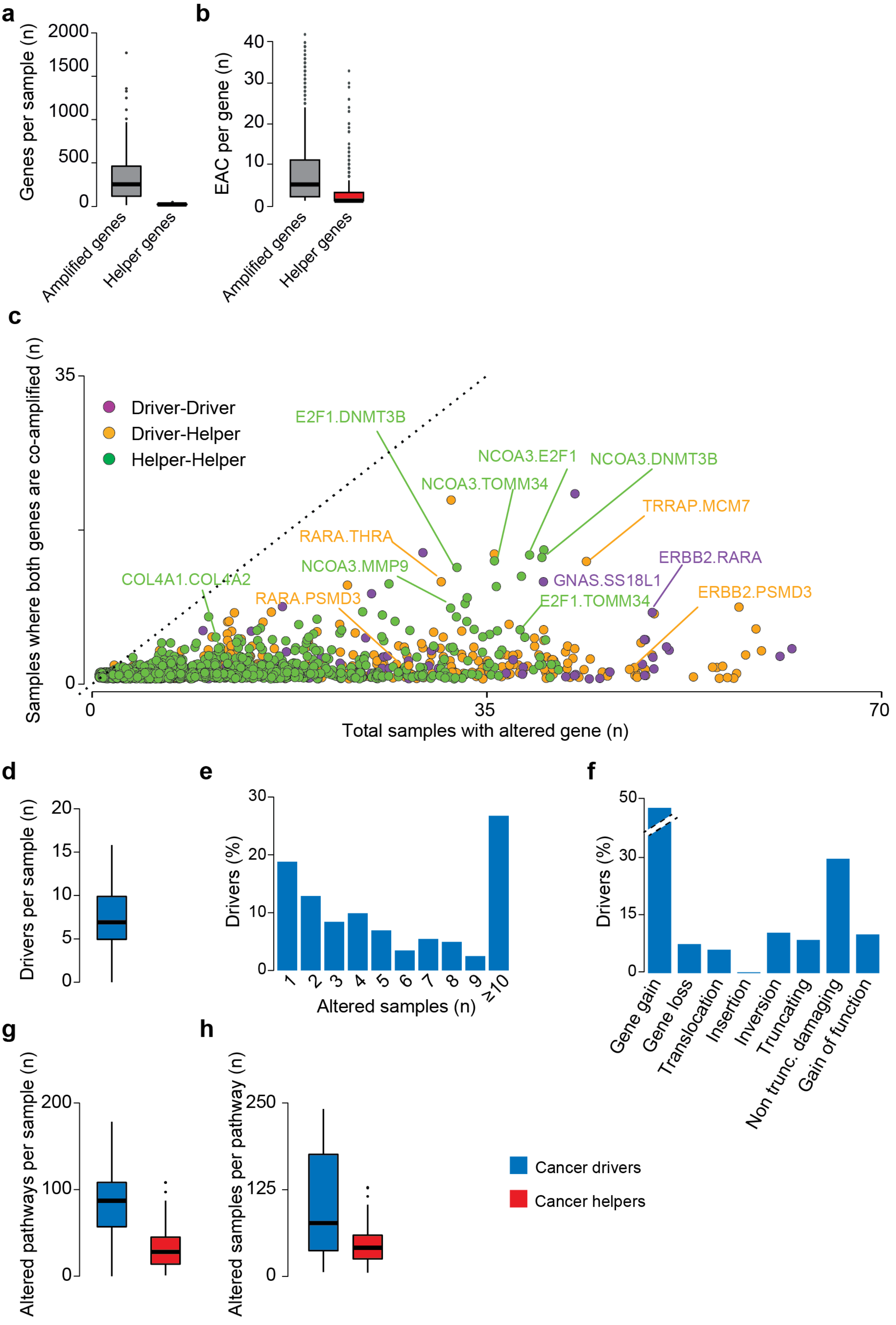

**a.** Distribution of 79,261 amplified genes and 2,062 amplified helper genes across 261 EACs. **b.** Distribution of 6,296 amplifications in the 952 genes across 261 EACs. Only 2,062 times (corresponding to 250 EACs) are these genes considered helpers. **c.** Co-amplified driver and helper genes in 261 EACs as a function of the total number of samples where they are altered. For each pair of drivers or helpers in the same chromosome, ASCAT breakpoints were used to assign whether they were in the same co-amplified segment. Only 1,345 gene pairs co-amplified in at least one sample are shown. The pairs TP53-SLC2A4 and TP53-SENP3 are co-amplified in 1 sample and altered in 197 samples are not shown. **d.** Distribution of known drivers across 261 EACs. Mean (n = 7.5) and median (n = 7) of the distribution are consistent with recent reports[7]. **e.** Recurrence of cancer drivers across 261 EACs. Only samples acquiring alterations with a damaging effect are considered. **f.** Distribution of damaging alterations in 202 cancer drivers. Overall, these genes acquire 1,967 damaging alterations. Distribution of altered pathways (**g**) and altered samples (**h**) for known drivers and newly predicted helpers. Lower and upper hinges and middle line of boxplots correspond to $25^{th}$, 75th and $50^{th}$ percentiles. Upper and lower whiskers extend less than 1.5 times the interquartile range.

**Supplementary Figure 5.** EAC clustering using pathways enriched in known drivers

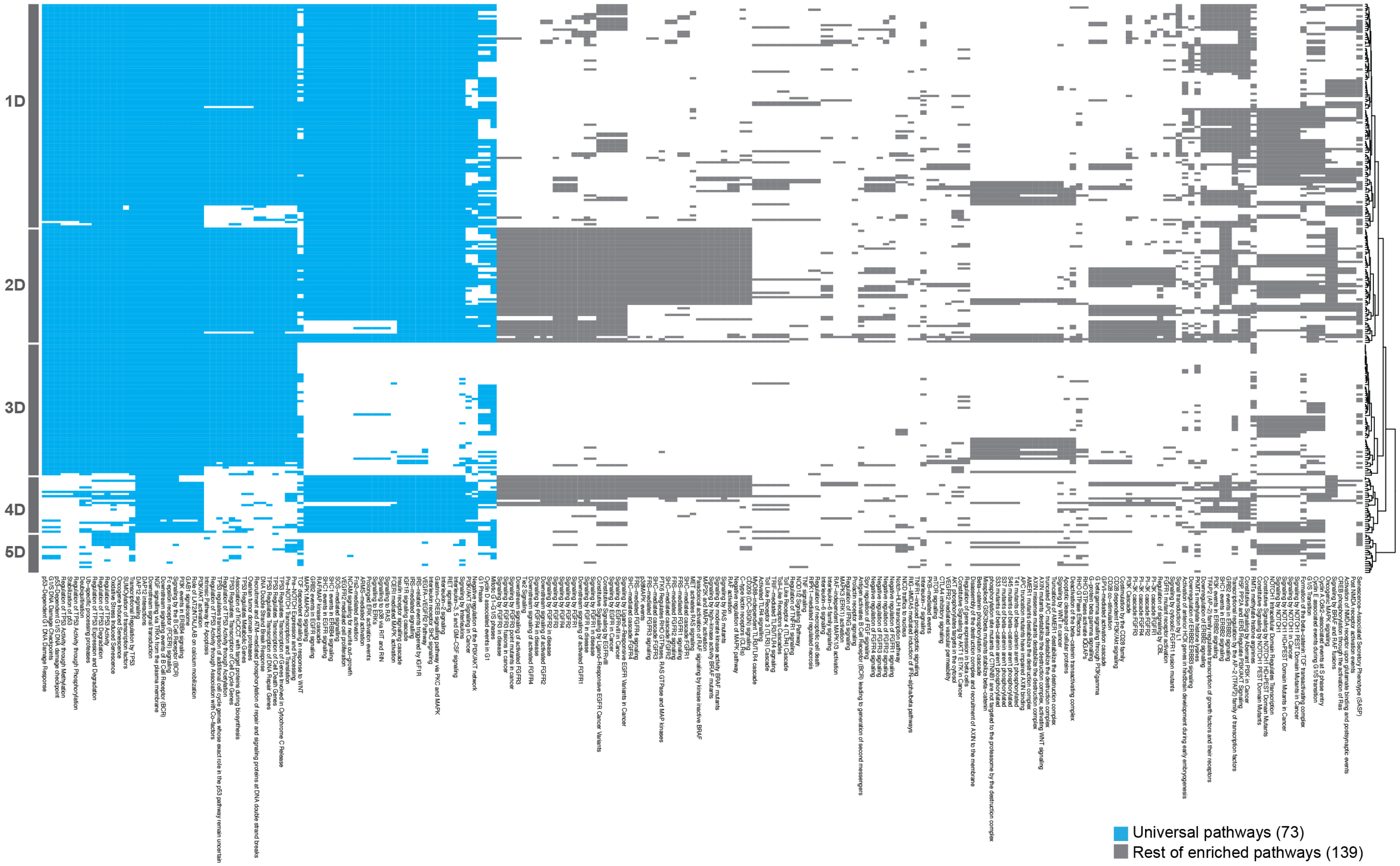

**Legend:** Hierarchical clustering was performed as described in Methods and Figure 2B and corresponds to that shown in Figure 2C for drivers, including the five clusters (1D-5D). Each row represents a sample and each column an enriched pathway. Samples were assigned to a given pathway if they had at least one altered known driver mapping to that pathway. Seventy-three universal pathways perturbed in at least 50% of samples are coloured in light blue.

**Supplementary Figure 6.** Identification of the optimal number of clusters

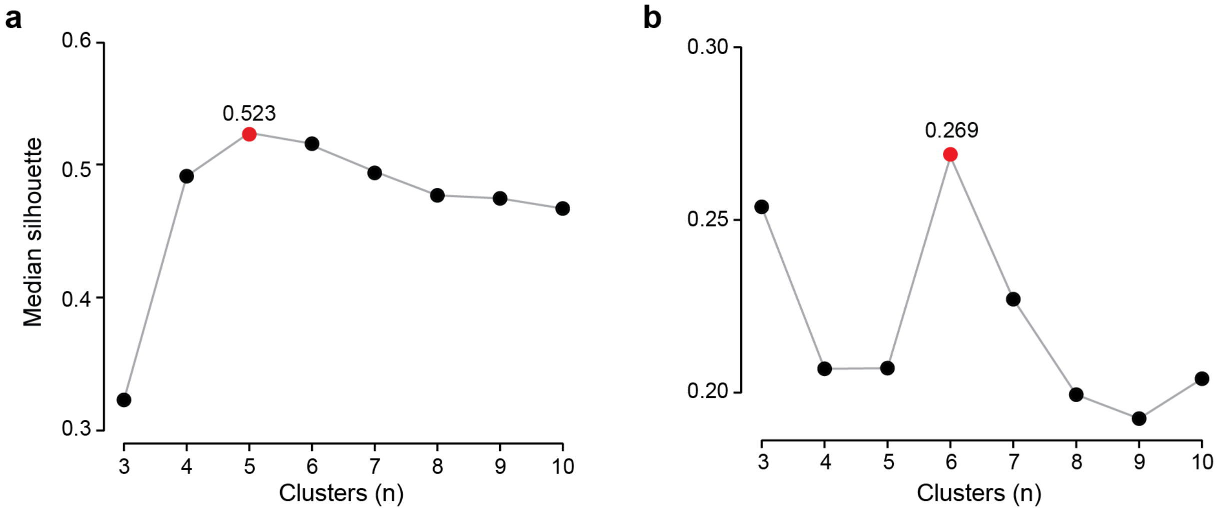


**Legend:** Silhouette analysis to measure clustering robustness of **(a)** known drivers and **(b)** helper genes. For each number of clusters between 3 and 10, clusters were derived from the dendrogram (Figure 2C) and the silhouette value[8] was then calculated for each sample using the Euclidean distance between rows of the Jaccard matrix $A_{ij}$. The number of clusters with the highest median silhouette value over all samples was chosen as the most robust clustering partition.

**Supplementary Figure 7.** EAC clustering using pathways enriched in helpers

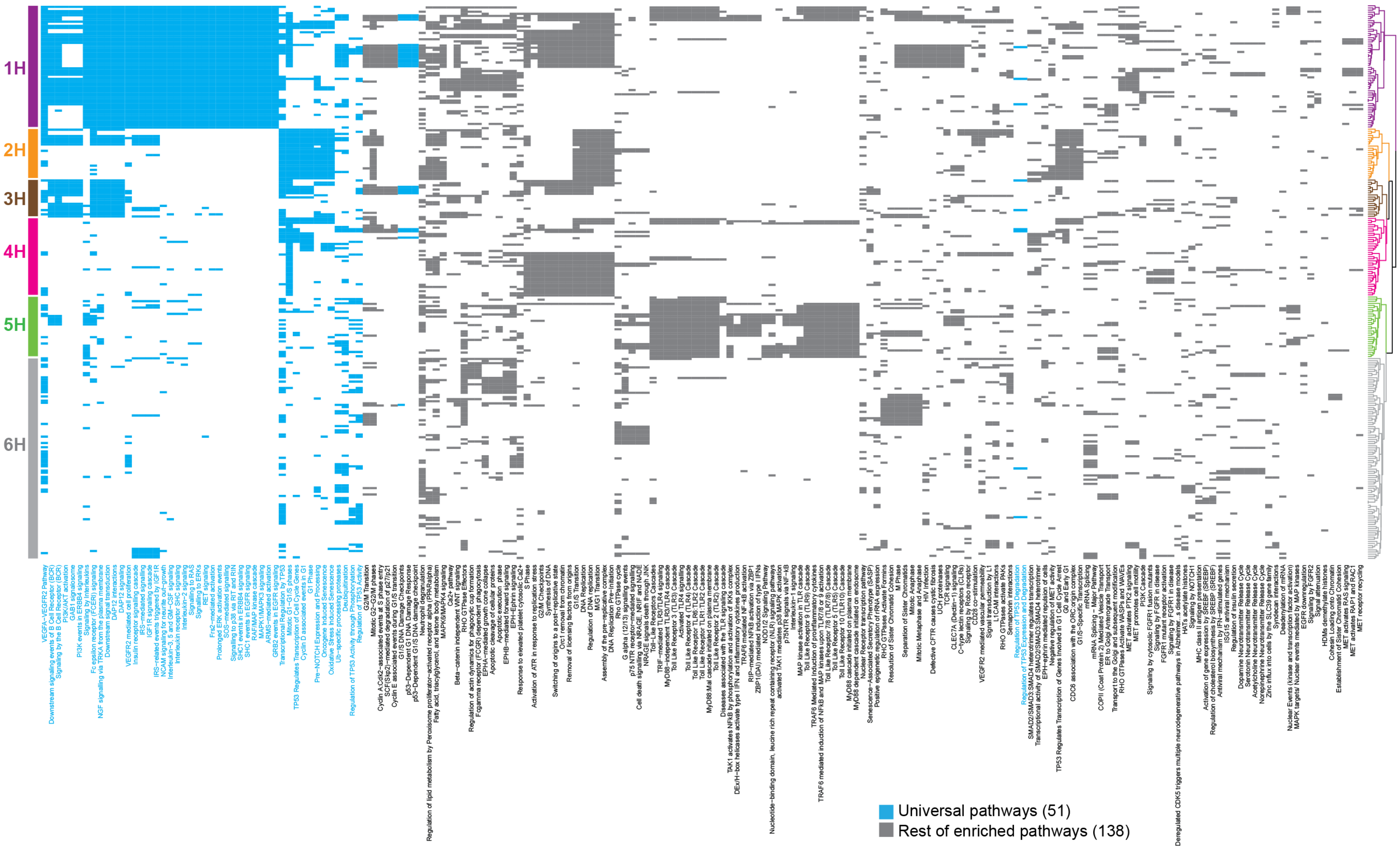

**Legend:** Hierarchical clustering was performed as described in Methods and Figure 2B and corresponds to that shown in Figure 2C for helpers, including the six clusters (1H-6H). Samples were assigned to a given pathway if they had at least one altered helper mapping to that pathway. Fifty-one of the 73 universal pathways perturbed in at least 50% of EACs are coloured in light blue. All other colours depict cluster-defining pathways**.**

**Supplementary Figure 8.** Cancer predisposition and comparison of helper genes

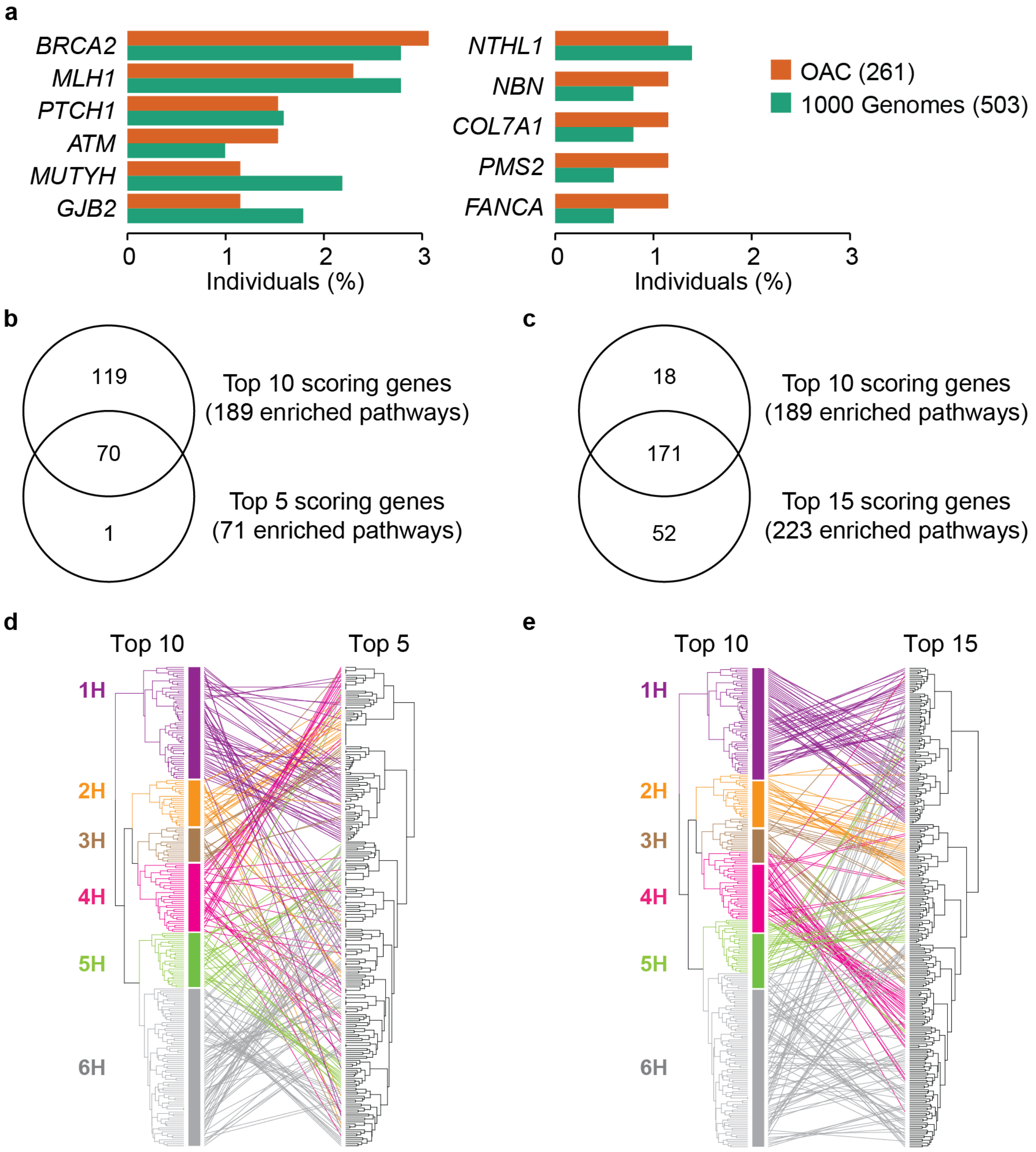


**Legend: a.** Germline rare damaging variants in cancer predisposition genes in 261 EAC patients and 503 Europeans from the 1000 Genomes Project. Of the 54 mutated predisposition genes, the ones altered in at least 1% of EAC patients (n=3) are shown. Fisher testing did not reveal any gene to be enriched in EAC patients compared to 1000 Genomes samples. Comparison of enriched pathways between top ten and (**b**) top five or (**c**) top 15 scoring genes in each sample. Gene set enrichment analysis using top five and top 15 scoring genes led to 71 and 223 enriched pathways,

respectively (FDR <0.01). Comparison of sample positions in the clustering dendrgrams of top 10 and (**d**) top five or (**e**) top 15 scoring genes in each sample. Complete linkage hierarchical clustering with Euclidean distance was used to group 261 EACs according to pathways enriched in the different datasets of helpers. The dendrogram of top 10 scoring genes corresponds to that shown in Figure 2C.

**Supplementary Figure 9.** Flow cytometry gating strategy and pseudocolour plots

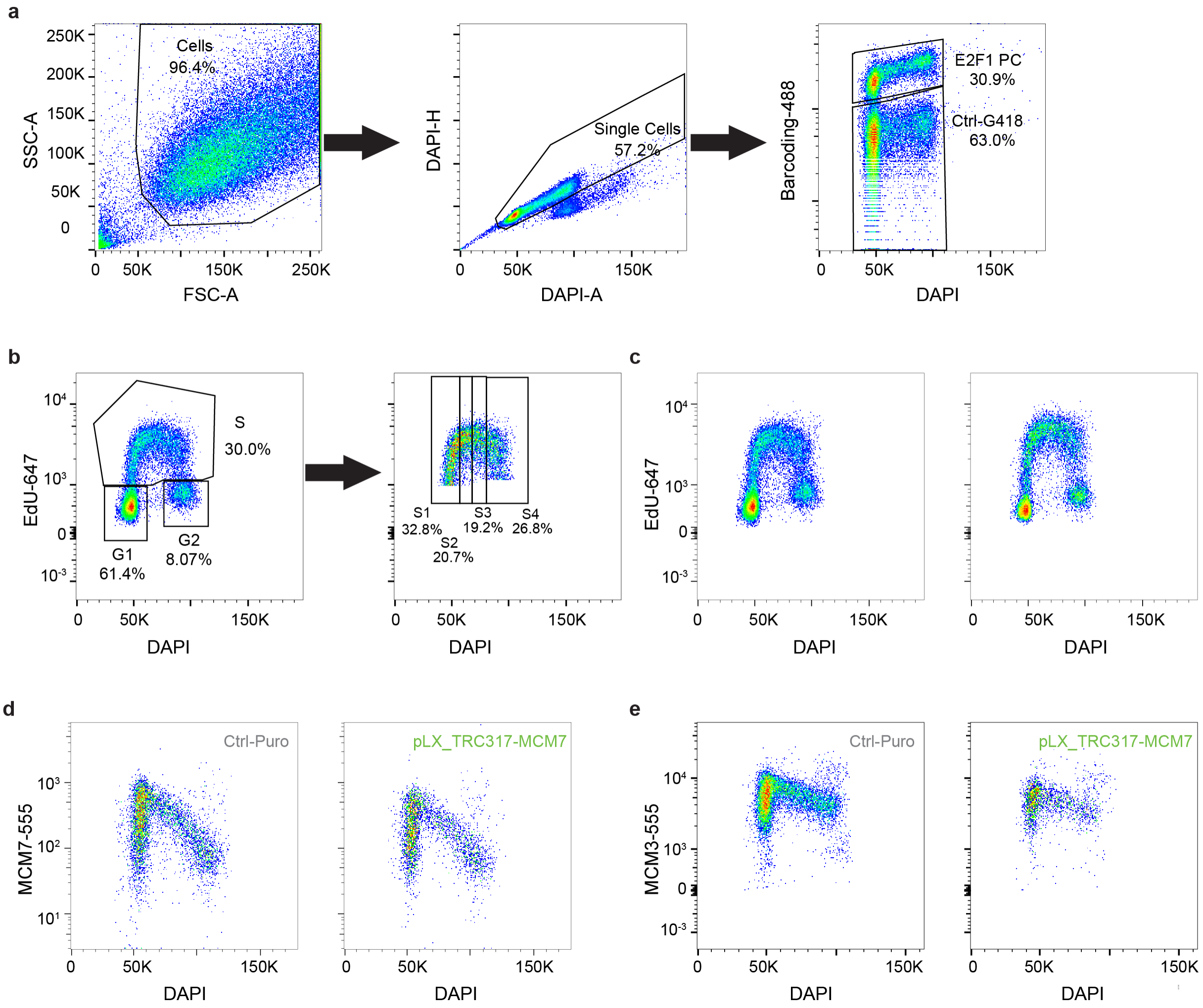


**Legend: a.** Firstly, cells were separated from debris using SSC-A and FSC-A. Secondly, single cells were separated from doublets by plotting DAPI-H against DAPI-A. Thirdly, barcoded populations within the same sample were gated to separate them, using 488-A plotted against DAPI-A. **b.** Cell cycle gates were made by plotting EdU-647-A against DAPI-A and separating G1, S and G2 populations. Then, S phase cells were further gated into four gates, called S1-S4. Cell cycle gates were copied exactly for each cell population within the sample. **c**,**d**,**e.** Pseudocolour plots corresponding to those in Figure 4D, E and F respectively. For each plot, 8000 events are shown. For each gate, the percentage of cells in the gate is shown.

**Supplementary Table 1. Description of sysSVM features.**

| Gene property | Feature for classification | Type | Cancer gene feature | Operational definition | Genes |
|---|---|---|---|---|---|
| Copy number variation | Gene gain | Molecular (categorical) | NA | CN >= 2*sample ploidy | 13,622 (79,216) |
| | Gene loss | Molecular (categorical) | NA | CN = 0 | 1,117 (3,089) |
| | Gene copy number (n) | Molecular (continuous) | NA | Somatic copy number (ASCAT) | 17,078 (116,989) |
| Structural variation | Gene translocation | Molecular (categorical) | NA | Somatic translocation event (Manta) | 5,577 (11,137) |
| | Gene inversion | Molecular (categorical) | NA | Somatic inversion event (Manta) | 5,546 (10,320) |
| | Gene insertion | Molecular (categorical) | NA | Somatic insertion event (Manta) | 519 (646) |
| SNVs and indels | Truncating alterations (n) | Molecular (continuous) | NA | Stopgain, stoploss, frameshift alterations (ANNOVAR) | 1,992 (2,471) |
| | Non-truncating damaging alterations (n) | Molecular (continuous) | NA | Damaging non-frameshift, nonsynonymous, splicing alterations (dbNSFP) | 7,287 (15,508) |
| | Gain of function alterations (n) | Molecular (continuous) | NA | Gain of function (OncodriveClust) | 170 (614) |
| | All exonic SNVs and indels (n) | Molecular (continuous) | NA | Silent and non-silent alterations (ANNOVAR) | 8,359 (18,941) |
| Gene length | Gene length (l) | Systems-level (continuous) | CGs tend to be long[1] | Length of the longest isoform (RefSeq) | 17,078 |
| Gene duplication | Gene duplication status | Systems-level (categorical) | TSGs are enriched in single-copy genes[9] | Mapping on >1 gene locus for ≥60% of protein length | 17,078 |
| Whole genome duplication | Ohnolog | Systems-level (categorical) | OGs are enriched in ohnologs[10] | Gene duplicate retained after whole genome duplications | 17,078 |
| Protein domains | Protein domains (n) | Systems-level (continuous) | CGs are enriched in multi-domain proteins[11] | Number of protein domains (CDD) | 17,039 |
| Chromatin state | Chromatin state | Systems-level (continuous) | CGs localise preferentially in open chromatin[12] | Chromatin state from Hi-C experiment in K562 cells. | 14,959 |
| Protein-protein interaction network | Protein degree (n) | Systems-level (continuous) | CGs encode preferentially protein hubs[9] | Number of connections in the protein-protein interaction network | 13,268 |
| | Hub | Systems-level (categorical) | | Top 25% most connected proteins | 3,398 |
| | Protein betweenness (n) | Systems-level (continuous) | CGs encode preferentially central proteins[9] | Centrality in the protein-protein interaction network | 13,268 |
| | Central protein | Systems-level (categorical) | | Top 25% most central proteins | 3,361 |

| | | | | | |
|---|---|---|---|---|---|
| miRNA interaction network | miRNA interactions (n) | Systems-level (continuous) | CGs tend to be regulated by a larger number of miRNAs[10] | Number of miRNAs interacting with the gene | 10,689 |
| Evolutionary origin | Old gene | Systems-level (categorical) | TSGs are enriched in old genes and OGs are enriched in genes originated in Metazoans[10] | The gene originated before metazoans | 10,493 |
| | Origin in prokaryotes | Systems-level (categorical) | | Oldest ortholog found in prokaryotes | 3,646 |
| | Origin in single cell eukaryotes | Systems-level (categorical) | | Oldest ortholog found in eukaryotes | 6,605 |
| | Origin in opisthokonts | Systems-level (categorical) | | Oldest ortholog found in opisthokonts | 242 |
| | Origin in metazoans | Systems-level (categorical) | | Oldest ortholog found in metazoans | 2,738 |
| | Origin in vertebrates | Systems-level (categorical) | | Oldest ortholog found in vertebrates | 2,003 |
| | Origin in mammals | Systems-level (categorical) | | Oldest ortholog found in mammals | 1,010 |
| | Origin in primates | Systems-level (categorical) | | Oldest ortholog found in primates | 110 |
| Expression | Ubiquitously expressed | Systems-level (categorical) | CGs are enriched in genes ubiquitously expressed[1,11] | Gene is expressed in ≤29/30 tissues | 11,052 |
| | Medium expressed | Systems-level (categorical) | | Gene is expressed in 3-28 tissues | 3,819 |
| | Selectively expressed | Systems-level (categorical) | | Gene is expressed in 2-3 tissues | 614 |
| | Specifically expressed | Systems-level (categorical) | | Gene is expressed in 1 tissue | 682 |
| | Not expressed | Systems-level (categorical) | | Gene is expressed in 0 tissues | 561 |
| | Tissues where the gene is expressed (n) | Systems-level (continuous) | | Number of tissues | 16,728 |

**Legend:** Listed are 10 molecular and 24 systems-level features used in sysSVM. For each of them, described are: the original gene property, whether it is categorical or continuous, its operational definition (see Methods) and the number of unique and redundant (in brackets) genes in 261 EACs. The description of systems-level properties of cancer genes is also given. For all systems-level properties, except gene length, duplication status and ohnologs, the number of unique genes before imputation is given (see Methods). CG = cancer gene; TSG = tumour suppressor gene; OG = oncogene; WGD = whole genome duplication; n = number; l = length.

**Supplementary Table 2:** Selection of best models and final list of helper genes

| Best model (n) | Linear kernel | Radial kernel | | Sigmoid kernel | | Polynomial kernel | | | List of top 10 genes | | Occurrence over 500 | |
|---|---|---|---|---|---|---|---|---|---|---|---|---|
| | nu | nu | $\gamma$ | nu | $\gamma$ | nu | $\gamma$ | degree | n | ID | times (n) | % |
| 1 | 0,05 | 0,05 | 0,03 | 0,05 | 4 | 0,05 | 0,03 | 3 | 1 | 952_A | 207 | 41,4 |
| 2 | 0,05 | 0,05 | 0,03 | 0,05 | 4 | 0,05 | 0,06 | 3 | 22 | 952_C | 1 | 0,2 |
| 2 | 0,05 | 0,05 | 0,03 | 0,05 | 4 | 0,05 | 0,06 | 3 | 2 | 952_B | 161 | 32,2 |
| 3 | 0,05 | 0,05 | 0,03 | 0,05 | 4 | 0,05 | 0,13 | 3 | 2 | 952_B | 161 | 32,2 |
| 3 | 0,05 | 0,05 | 0,03 | 0,05 | 4 | 0,05 | 0,13 | 3 | 5 | 951_B | 19 | 3,8 |
| 4 | 0,05 | 0,05 | 0,03 | 0,05 | 4 | 0,05 | 0,25 | 3 | 2 | 952_B | 161 | 32,2 |
| 4 | 0,05 | 0,05 | 0,03 | 0,05 | 4 | 0,05 | 0,25 | 3 | 5 | 951_B | 19 | 3,8 |
| 5 | 0,05 | 0,05 | 0,03 | 0,05 | 4 | 0,05 | 1,00 | 3 | 2 | 952_B | 161 | 32,2 |
| 6 | 0,05 | 0,05 | 0,03 | 0,05 | 4 | 0,05 | 4,00 | 3 | 2 | 952_B | 161 | 32,2 |
| 7 | 0,05 | 0,05 | 0,03 | 0,05 | 4 | 0,05 | 8,00 | 3 | 2 | 952_B | 161 | 32,2 |
| 8 | 0,05 | 0,05 | 0,03 | 0,05 | 4 | 0,05 | 16,00 | 3 | 2 | 952_B | 161 | 32,2 |
| 9 | 0,05 | 0,05 | 0,03 | 0,05 | 4 | 0,05 | 0,02 | 3 | 3 | 951_A | 43 | 8,6 |
| 9 | 0,05 | 0,05 | 0,03 | 0,05 | 4 | 0,05 | 0,02 | 3 | 10 | 950 | 3 | 0,6 |
| 10 | 0,05 | 0,05 | 0,03 | 0,05 | 8 | 0,05 | 0,02 | 3 | 23 | 934_B | 1 | 0,2 |
| 10 | 0,05 | 0,05 | 0,03 | 0,05 | 8 | 0,05 | 0,02 | 3 | 4 | 934_A | 28 | 5,6 |
| 11 | 0,05 | 0,05 | 0,03 | 0,05 | 8 | 0,05 | 0,03 | 3 | 4 | 934_A | 28 | 5,6 |
| 12 | 0,05 | 0,05 | 0,03 | 0,05 | 8 | 0,05 | 0,06 | 3 | 4 | 934_A | 28 | 5,6 |
| 13 | 0,05 | 0,05 | 0,03 | 0,05 | 8 | 0,05 | 0,25 | 3 | 4 | 934_A | 28 | 5,6 |
| 14 | 0,05 | 0,05 | 0,03 | 0,05 | 8 | 0,05 | 0,50 | 3 | 4 | 934_A | 28 | 5,6 |
| 15 | 0,05 | 0,05 | 0,03 | 0,05 | 8 | 0,05 | 1,00 | 3 | 4 | 934_A | 28 | 5,6 |
| 16 | 0,05 | 0,05 | 0,03 | 0,05 | 8 | 0,05 | 16,00 | 3 | 4 | 934_A | 28 | 5,6 |
| 17 | 0,05 | 0,05 | 0,03 | 0,05 | 4 | 0,05 | 0,50 | 3 | 5 | 951_B | 19 | 3,8 |
| 18 | 0,05 | 0,05 | 0,03 | 0,05 | 4 | 0,05 | 2,00 | 3 | 5 | 951_B | 19 | 3,8 |
| 19 | 0,05 | 0,05 | 0,03 | 0,05 | 16 | 0,05 | 0,06 | 3 | 6 | 929_A | 8 | 1,6 |
| 20 | 0,05 | 0,05 | 0,03 | 0,05 | 16 | 0,05 | 0,25 | 3 | 6 | 929_A | 8 | 1,6 |
| 20 | 0,05 | 0,05 | 0,03 | 0,05 | 16 | 0,05 | 0,25 | 3 | 18 | 931 | 1 | 0,2 |
| 21 | 0,05 | 0,05 | 0,03 | 0,05 | 16 | 0,05 | 2,00 | 3 | 6 | 929_A | 8 | 1,6 |
| 22 | 0,05 | 0,05 | 0,03 | 0,05 | 16 | 0,05 | 4,00 | 3 | 6 | 929_A | 8 | 1,6 |
| 23 | 0,05 | 0,05 | 0,03 | 0,05 | 16 | 0,05 | 16,00 | 3 | 6 | 929_A | 8 | 1,6 |
| 24 | 0,05 | 0,05 | 0,03 | 0,05 | 2 | 0,05 | 0,02 | 3 | 21 | 916 | 1 | 0,2 |
| 24 | 0,05 | 0,05 | 0,03 | 0,05 | 2 | 0,05 | 0,02 | 3 | 7 | 915 | 5 | 1 |
| 25 | 0,05 | 0,05 | 0,03 | 0,05 | 2 | 0,05 | 0,25 | 3 | 7 | 915 | 5 | 1 |
| 26 | 0,05 | 0,05 | 0,02 | 0,05 | 4 | 0,05 | 0,02 | 3 | 9 | 929_B | 3 | 0,6 |
| 26 | 0,05 | 0,05 | 0,02 | 0,05 | 4 | 0,05 | 0,02 | 3 | 18 | 931 | 1 | 0,2 |
| 26 | 0,05 | 0,05 | 0,02 | 0,05 | 4 | 0,05 | 0,02 | 3 | 8 | 928 | 4 | 0,8 |
| 27 | 0,05 | 0,05 | 0,02 | 0,05 | 4 | 0,05 | 8,00 | 3 | 8 | 928 | 4 | 0,8 |
| 28 | 0,05 | 0,1 | 0,02 | 0,05 | 16 | 0,05 | 0,25 | 3 | 11 | 920 | 3 | 0,6 |
| 29 | 0,05 | 0,05 | 0,02 | 0,05 | 16 | 0,05 | 0,02 | 3 | 12 | 926_A | 2 | 0,4 |
| 30 | 0,05 | 0,05 | 0,01 | 0,05 | 2 | 0,05 | 0,25 | 3 | 13 | 898 | 1 | 0,2 |
| 31 | 0,05 | 0,05 | 0,01 | 0,05 | 16 | 0,05 | 0,25 | 3 | 14 | 911 | 1 | 0,2 |
| 32 | 0,05 | 0,05 | 0,02 | 0,05 | 0,5 | 0,05 | 0,06 | 3 | 15 | 907 | 1 | 0,2 |
| 33 | 0,05 | 0,05 | 0,02 | 0,05 | 1 | 0,05 | 0,06 | 3 | 16 | 909 | 1 | 0,2 |
| 34 | 0,05 | 0,05 | 0,02 | 0,05 | 2 | 0,05 | 0,25 | 3 | 17 | 906 | 1 | 0,2 |
| 35 | 0,05 | 0,05 | 0,02 | 0,05 | 8 | 0,05 | 0,02 | 3 | 19 | 926_B | 1 | 0,2 |
| 36 | 0,05 | 0,05 | 0,03 | 0,05 | 0,5 | 0,05 | 0,02 | 3 | 17 | 906 | 1 | 0,2 |
| 37 | 0,05 | 0,05 | 0,03 | 0,05 | 1 | 0,05 | 0,25 | 3 | 20 | 919 | 1 | 0,2 |
| 38 | 0,05 | 0,1 | 0,02 | 0,05 | 2 | 0,05 | 0,02 | 3 | 24 | 908 | 1 | 0,2 |

**Legend:** Shown are the parameters of the 38 unique best models in the four kernels and 24 associated unique lists of top 10 genes. These lists are named using the number of genes that compose them, followed by a letter where the same number (but not the same genes) was found multiple times. The number of times and corresponding

frequency that each set of best models and list of top 10 genes was found over 500 sets and lists are also shown.

**Supplementary Table 3:** List of oligos used in the study

| Experiment | Gene/ Protein | Oligo | Sequence | Protein position |
|---|---|---|---|---|
| CRISPR gene editing | ABI2 (Q9NYB9) | ABI2_crRNA1 (Sigma-Aldrich) | GGCAACACTTGCTAAGGAT | S57-A62 |
| | | ABI2_crRNA2 (Sigma-Aldrich) | GCCTATCTGATAAACACCT | A62-T67 |
| | | ABI2_crRNA3 (Sigma-Aldrich) | AGATTCCATCCTTCGTAGC | Q82-S88 |
| | | ABI2-203366920 (Synthego) | UGCUAAGGAUUGGGUGGUGU | Y53-A59 |
| | | ABI2-203366926 (Synthego) | AACACUUGCUAAGGAUUGGG | T55-V61 |
| | NCOR2 (Q9Y618) | NCOR2_crRNA1 (Sigma-Aldrich) | TCGCTGCGGGCGGCCGACA | L361-H370 |
| | | NCOR2_crRNA2 (Sigma-Aldrich) | ACCCGCTCAATGGCTAATG | R581-A590 |
| | | NCOR2_crRNA3 (Sigma-Aldrich) | ACAGCGCCATCACATACCG | S1227-G1235 |
| | | NCOR2-124486566 (Synthego) | GUCCCCUCCUGCAGGACGUC | T35-V37 |
| | | NCOR2-124486567 (Synthego) | UGUCCCCUCCUGCAGGACGU | T35-V38 |
| | TP53 (P04637) | TP53+7676265 (Synthego) | CCAUUGCUUGGGACGGCAAG | P34-D41 |
| | | TP53+7676266 (Synthego) | CAUUGCUUGGGACGGCAAGG | P34-M40 |
| | Non Target Control (NTC) | NTC_crRNA1 | GATACGTCGGTACCGGACCG | NA |
| | | NTC_crRNA2 | GTAACGCGAACTACGCGGGT | NA |
| | | NTC_crRNA3 | GTCGACGTTATTGCCGGTCG | NA |
| | | NTC_crRNA4 | GGAAACCTACGTCGACGAAT | NA |
| | | NTC_crRNA5 | GCTCTCGTACGGCGCGTATC | NA |
| MiSeq | NCOR2 | NCOR2_forward1 | TCGTCGGCAGCGTCAGATGTGTATAAGAGACAGCCCTCGACGTAAACCACCC | NA |
| | | NCOR2_reverse1 | GTCTCGTGGGCTCGGAGATGTGTATAAGAGACAGCCACACTTCTCCTCTGGGG | NA |
| | | NCOR2_forward2 | TCGTCGGCAGCGTCAGATGTGTATAAGAGACAGCAGTAGGTAGCGCTGGGATT | NA |
| | | NCOR2_reverse2 | GTCTCGTGGGCTCGGAGATGTGTATAAGAGACAGAAGACAGACGACACCTCAGG | NA |
| | | NCOR2_forward3 | TCGTCGGCAGCGTCAGATGTGTATAAGAGACAGAGGGGTTATAAGATGGGCTGG | NA |
| | | NCOR2_reverse3 | GTCTCGTGGGCTCGGAGATGTGTATAAGAGACAGGACTCCCTCTGCGTTGAAAC | NA |

| | | | | |
|---|---|---|---|---|
| | | NCOR2_forward4 | TCGTCGGCAGCGTCAGATGTGTATAAGAGACAGTCTCCTCACCGTTCATTCCC | NA |
| | | NCOR2_reverse4 | GTCTCGTGGGCTCGGAGATGTGTATAAGAGACAGTGCAGGACTTGGGCTTATCT | NA |
| | ABI2 | ABI2_forward1 | TCGTCGGCAGCGTCAGATGTGTATAAGAGACAGTGGACTCAGCAGAATCGTTG | NA |
| | | ABI2_reverse1 | GTCTCGTGGGCTCGGAGATGTGTATAAGAGACAGTGCCAGCATTACAGATAGCCT | NA |
| | | ABI2_forward2 | TCGTCGGCAGCGTCAGATGTGTATAAGAGACAGTGGACTCAGCAGAATCGTTG | NA |
| | | ABI2_reverse2 | GTCTCGTGGGCTCGGAGATGTGTATAAGAGACAGAGCTGGGATGCCTGGATATC | NA |
| | TP53 | TP53_forward | TCGTCGGCAGCGTCAGATGTGTATAAGAGACAGCTCTGGCATTCTGGGAGCTT | NA |
| | | TP53_reverse | GTCTCGTGGGCTCGGAGATGTGTATAAGAGACAGGAAGCGAAAATTCCATGGGAC | NA |
| Quantitative RT-PCR | MCM7 | MCM7_forward | ATCGGATTGTGAAGATGAAC | NA |
| | | MCM7_reverse | CTTTTCGTAGAAATCCTCCTC | NA |

**Legend:** Reported are the DNA and RNA sequences of the oligos used in this study. * = selected for knockdown experiment. NA = not applicable.

## Supplementary References


1. D'Antonio M, Ciccarelli FD. Integrated analysis of recurrent properties of cancer genes to identify novel drivers. *Genome Biol* **14**, R52 (2013).
2. Lawrence MS*, et al.* Mutational heterogeneity in cancer and the search for new cancer-associated genes. *Nature* **499**, 214-218 (2013).
3. Scholkopf B, Platt JC, Shawe-Taylor J, Smola AJ, Williamson RC. Estimating the support of a high-dimensional distribution. *Neural Comput* **13**, 1443-1471 (2001).
4. Meyer D*, et al.* e1071: Misc Functions of the Department of Statistics, Probability Theory Group (Formerly: E1071), TU Wien. (ed^(eds). R package version 1.6-8 edn (2017).
5. Guyon I, Weston J, Barnhill S, Vapnik V. Gene Selection for Cancer Classification using Support Vector Machines. *Machine learning* **46**, 389-422 (2002).
6. Nones K*, et al.* Genomic catastrophes frequently arise in esophageal adenocarcinoma and drive tumorigenesis. *Nat Commun* **5**, 5224 (2014).
7. Martincorena I*, et al.* Universal Patterns of Selection in Cancer and Somatic Tissues. *Cell* **171**, 1029-1041 e1021 (2017),Sabarinathan R*, et al.* The whole-genome panorama of cancer drivers. *bioRxiv*, (2017).
8. Rousseeuw PJ. Silhouettes: A graphical aid to the interpretation and validation of cluster analysis. *Journal of Computational and Applied Mathematics* **20**, (1987).
9. Rambaldi D, Giorgi FM, Capuani F, Ciliberto A, Ciccarelli FD. Low duplicability and network fragility of cancer genes. *Trends in Genetics* **24**, 427-430 (2008).
10. D'Antonio M, Ciccarelli FD. Modification of gene duplicability during the evolution of protein interaction network. *PLoS Comput Biol* **7**, e1002029 (2011).

11 An O, Dall'Olio GM, Mourikis TP, Ciccarelli FD. NCG 5.0: updates of a manually curated repository of cancer genes and associated properties from cancer mutational screenings. *Nucleic Acids Research* **44**, D992-D999. (2016).

12 Lieberman-Aiden E*, et al.* Comprehensive mapping of long-range interactions reveals folding principles of the human genome. *Science* **326**, 289-293 (2009).
