# Supplementary material for: Patient-specific cancer genes contribute to recurrently perturbed pathways and establish therapeutic vulnerabilities in esophageal adenocarcinoma
Source: Nat Commun. 2019 Jul 15;10:3101. doi: 10.1038/s41467-019-10898-3 (PMC6629660; doi:10.1038/s41467-019-10898-3)
Supplement: Supplementary file 3 — Description of Additional Supplementary Files [file 41467_2019_10898_MOESM3_ESM.pdf]

Description of Additional Supplementary Files

File Name: Supplementary Data 1
Description: **Description of EACs used in this study.** For each of the 261 ICGC, 86 TCGA, and 21 EACs from Nones et al, the number of genes with predicted damaging alterations is provided. The total number of altered genes is 116,989 (17,087 unique hits), 155,886 (17,779 unique hits), and 8,800 (5,617 unique hits) in the three datasets, respectively. After refinement of known cancer genes and exclusion of not expressed genes in esophagus according to GTEx v.4, genes considered for downstream analysis were 87,269 (12,771 unique hits), 116,218 (13,225 unique hits) and 6,479 (4,157 unique hits), respectively. For each sample, drivers and helpers and, for EACs in the ICGC cohort, also clinical information, enriched pathways and patient cluster are reported.

File Name: Supplementary Data 2
Description: **Known cancer genes with driver alterations.** List of 476 known cancer genes from the cancer gene census (7) that acquire potential damaging alterations in 261 EACs. For each gene, the number of samples with damaging alterations in the 261 EACs and the number of samples in which the gene was considered as a driver is reported. This was based on the manual assessment of (a) the literature support for the cancer driver role in EAC, (b) the role as a tumour suppressor or oncogene as reported in (8) and the type of alterations found in EACs. For the 202 genes considered as drivers in at least one sample, the corresponding enriched pathways are reported too.

File Name: Supplementary Data 3
Description: **Cancer helper genes in 261 EACs.** List of 952 cancer helper genes in the 261 EACs. For each gene reported are the gene description; the number and percentage of samples where it is altered; the type of alteration; whether it was recently found as a known driver or a candidate cancer gene in EAC (marked with *) or other cancer types through large scale sequencing screenings, as reported in the NCG database (90); and the associated enriched pathways. NA = not applicable.

File Name: Supplementary Data 4
Description: **Gene set enrichment analysis of EAC drivers or helpers.** Shown are the 212 and 189 pathways enriched in drivers and helpers (FDR <0.01, 76 are enriched in both). For each pathway, the number of genes and samples, the p-value of one-tailed hypergeometric test and the False Discovey Rate (FDR) using the Benjamini and Hochberg method are reported for both drivers and helpers. Pathway size refers to the total number of genes in the pathway. Universal pathways are those with at least one perturbed driver in at least 50% of samples.
