# Supplementary material for: Patient-specific cancer genes contribute to recurrently perturbed pathways and establish therapeutic vulnerabilities in esophageal adenocarcinoma
Source: Nat Commun. 2019 Jul 15;10:3101. doi: 10.1038/s41467-019-10898-3 (PMC6629660; doi:10.1038/s41467-019-10898-3)
Supplement: Supplementary file 8 — Reporting Summary [file 41467_2019_10898_MOESM8_ESM.pdf]

nature research

Corresponding author(s): Francesca D. Ciccarelli

Last updated by author(s): 22/03/2019

Reporting Summary

Nature Research wishes to improve the reproducibility of the work that we publish. This form provides structure for consistency and transparency in reporting. For further information on Nature Research policies, see Authors & Referees and the Editorial Policy Checklist.

Statistics

For all statistical analyses, confirm that the following items are present in the figure legend, table legend, main text, or Methods section.

n/a	Confirmed	
☒	☐	The exact sample size (*n*) for each experimental group/condition, given as a discrete number and unit of measurement
☐	☒	A statement on whether measurements were taken from distinct samples or whether the same sample was measured repeatedly
☐	☒	The statistical test(s) used AND whether they are one- or two-sided<br>*Only common tests should be described solely by name; describe more complex techniques in the Methods section.*
☒	☐	A description of all covariates tested
☐	☒	A description of any assumptions or corrections, such as tests of normality and adjustment for multiple comparisons
☐	☒	A full description of the statistical parameters including central tendency (e.g. means) or other basic estimates (e.g. regression coefficient) AND variation (e.g. standard deviation) or associated estimates of uncertainty (e.g. confidence intervals)
☐	☒	For null hypothesis testing, the test statistic (e.g. *F*, *t*, *r*) with confidence intervals, effect sizes, degrees of freedom and *P* value noted<br>*Give P values as exact values whenever suitable.*
☒	☐	For Bayesian analysis, information on the choice of priors and Markov chain Monte Carlo settings
☐	☒	For hierarchical and complex designs, identification of the appropriate level for tests and full reporting of outcomes
☒	☐	Estimates of effect sizes (e.g. Cohen's *d*, Pearson's *r*), indicating how they were calculated

Our web collection on statistics for biologists contains articles on many of the points above.

Software and code

Policy information about availability of computer code

Data collection	Data sources: International Cancer Genome Consortium, The Cancer Genome Atlas, previous studies (see main text)
Data analysis	All code used in this study was written in R programming language and scripts are available upon request.

For manuscripts utilizing custom algorithms or software that are central to the research but not yet described in published literature, software must be made available to editors/reviewers. We strongly encourage code deposition in a community repository (e.g. GitHub). See the Nature Research guidelines for submitting code & software for further information.

Data

Policy information about availability of data

All manuscripts must include a data availability statement. This statement should provide the following information, where applicable:

- Accession codes, unique identifiers, or web links for publicly available datasets
- A list of figures that have associated raw data
- A description of any restrictions on data availability

The WGS and RNA sequencing data can be accessed at the European Genome-phenome Archive with the accession numbers EGAD00001004775 and EGAD00001004776, respectively. Source data for figures 4-6 are provided as a Source Data file.

Field-specific reporting

Please select the one below that is the best fit for your research. If you are not sure, read the appropriate sections before making your selection.

☒ Life sciences ☐ Behavioural & social sciences ☐ Ecological, evolutionary & environmental sciences

Life sciences study design

All studies must disclose on these points even when the disclosure is negative.

Sample size	No statistical approach was used to pre-determine the sample size
Data exclusions	No data were excluded
Replication	Findings in this study were reproduced in independent cohorts of esophageal adenocarcinomas.
Randomization	Randomization was used during cross-validation (see Methods).
Blinding	N/A

Reporting for specific materials, systems and methods

We require information from authors about some types of materials, experimental systems and methods used in many studies. Here, indicate whether each material, system or method listed is relevant to your study. If you are not sure if a list item applies to your research, read the appropriate section before selecting a response.

Materials & experimental systems

n/a	Involved in the study	
☐	☒	Antibodies
☐	☒	Eukaryotic cell lines
☒	☐	Palaeontology
☒	☐	Animals and other organisms
☐	☒	Human research participants
☐	☒	Clinical data

Methods

n/a	Involved in the study	
☒	☐	ChIP-seq
☐	☒	Flow cytometry
☒	☐	MRI-based neuroimaging

Antibodies

Antibodies used	Mouse monoclonal anti-MCM7 Santa Cruz Biotechnology Sc-56324<br>Rabbit polyclonal Anti-MCM3 Bethyl A300-192A<br>Alexa Fluor 555-conjugated donkey anti-mouse Thermo Fisher A-31570<br>Alexa Fluor 555-conjugated donkey anti-rabbit Thermo Fisher A-31572
Validation	All antibodies are commercially available and validated

Eukaryotic cell lines

Policy information about cell lines

Cell line source(s)	FLO-1 cells from ECACC General Cell Collection Catalogue no. 11012001<br>OE19 cells from The Francis Crick Institute cell services<br>OE33 cells from ECACC General Cell Collection Catalogue no. 96070808<br>MFD-1 cells from the OCCAMS Consortium<br>CP-A (KR-42421) cells from ATCC Catalogue no. CRL-4027
Authentication	Cells were validated by short tandem repeat analysis
Mycoplasma contamination	Cells were routinely tested for mycoplasma
Commonly misidentified lines<br>(See ICLAC register)	N/A

Human research participants

Policy information about studies involving human research participants

Population characteristics	Tumour sequencing data from male and female patients (40-87 years old) diagnosed with esophageal adenocarcinoma were used for this study.

Recruitment | No patients were recruited specifically for this study. Samples used were cohorts of patients previously published.

Ethics oversight | *Identify the organization(s) that approved the study protocol.*

Note that full information on the approval of the study protocol must also be provided in the manuscript.

Clinical data

Policy information about clinical studies

All manuscripts should comply with the ICMJE guidelines for publication of clinical research and a completed CONSORT checklist must be included with all submissions.

Clinical trial registration	*Provide the trial registration number from ClinicalTrials.gov or an equivalent agency.*
Study protocol	*Note where the full trial protocol can be accessed OR if not available, explain why.*
Data collection	*Describe the settings and locales of data collection, noting the time periods of recruitment and data collection.*
Outcomes	*Describe how you pre-defined primary and secondary outcome measures and how you assessed these measures.*

Flow Cytometry

Plots

Confirm that:

☒ The axis labels state the marker and fluorochrome used (e.g. CD4-FITC).

☒ The axis scales are clearly visible. Include numbers along axes only for bottom left plot of group (a 'group' is an analysis of identical markers).

☒ All plots are contour plots with outliers or pseudocolor plots.

☒ A numerical value for number of cells or percentage (with statistics) is provided.

Methodology

Sample preparation	Sample preparation listed in Methods
Instrument	BD LSR II Fortessa flow cytometer (BD Biosciences)
Software	Compensation was performed manually with single colour controls, using BD FACSDiva software (BD Biosciences). FlowJo 10.3 software was used to analyse MCM loading onto chromatin and EdU incorporation
Cell population abundance	1-3 million FLO-1 cancer cells were sorted per sample.
Gating strategy	The cells were then separated by gating the barcoded populations using 488-A/DAPI-A. Cells were finally separated into cell cycle gates (G1, S1-4, G2) based on EdU-647-A and DAPI-A (Supplementary Figure 9), and the geometric mean fluorescence intensity was obtained for each channel (MCM-555 or EdU-647)

☒ Tick this box to confirm that a figure exemplifying the gating strategy is provided in the Supplementary Information.
